# Patient outcomes after critical illness: a systematic review of qualitative studies following hospital discharge

**DOI:** 10.1186/s13054-016-1516-x

**Published:** 2016-10-26

**Authors:** Mohamed D. Hashem, Aparna Nallagangula, Swaroopa Nalamalapu, Krishidhar Nunna, Utkarsh Nausran, Karen A. Robinson, Victor D. Dinglas, Dale M. Needham, Michelle N. Eakin

**Affiliations:** 1 Outcomes After Critical Illness and Surgery Group, Johns Hopkins University, Baltimore, MD USA; 2 Division of Pulmonary and Critical Care Medicine, School of Medicine, Johns Hopkins University, Baltimore, MD USA; 3 Department of Medicine, School of Medicine, Johns Hopkins University, Baltimore, MD USA; 4 Department of Physical Medicine and Rehabilitation, School of Medicine, Johns Hopkins University, Baltimore, MD USA

**Keywords:** Critical illness, Qualitative research, Patient outcomes, Quality of life

## Abstract

**Background:**

There is growing interest in patient outcomes following critical illness, with an increasing number and different types of studies conducted, and a need for synthesis of existing findings to help inform the field. For this purpose we conducted a systematic review of qualitative studies evaluating patient outcomes after hospital discharge for survivors of critical illness.

**Methods:**

We searched the PubMed, EMBASE, CINAHL, PsycINFO, and CENTRAL databases from inception to June 2015. Studies were eligible for inclusion if the study population was >50 % adults discharged from the ICU, with qualitative evaluation of patient outcomes. Studies were excluded if they focused on specific ICU patient populations or specialty ICUs. Citations were screened in duplicate, and two reviewers extracted data sequentially for each eligible article. Themes related to patient outcome domains were coded and categorized based on the main domains of the Patient Reported Outcomes Measurement Information System (PROMIS) framework.

**Results:**

A total of 2735 citations were screened, and 22 full-text articles were eligible, with year of publication ranging from 1995 to 2015. All of the qualitative themes were extracted from eligible studies and then categorized using PROMIS descriptors: satisfaction with life (16 studies), including positive outlook, acceptance, gratitude, independence, boredom, loneliness, and wishing they had not lived; mental health (15 articles), including symptoms of post-traumatic stress disorder, anxiety, depression, and irritability/anger; physical health (14 articles), including mobility, activities of daily living, fatigue, appetite, sensory changes, muscle weakness, and sleep disturbances; social health (seven articles), including changes in friends/family relationships; and ability to participate in social roles and activities (six articles), including hobbies and disability.

**Conclusion:**

ICU survivors may experience positive emotions and life satisfaction; however, a wide range of mental, physical, social, and functional sequelae occur after hospital discharge. These findings are important for understanding patient-centered outcomes in critical care and providing focus for future interventional studies aimed at improving outcomes of importance to ICU survivors.

**Electronic supplementary material:**

The online version of this article (doi:10.1186/s13054-016-1516-x) contains supplementary material, which is available to authorized users.

## Background

With the aging population, the number of patients admitted to ICUs continues to grow, as does the number of ICU survivors [1, 2]. These survivors frequently have substantial morbidity after hospital discharge, including physical, cognitive, and mental health impairments [3–6], collectively known as post-intensive care syndrome (PICS) [7]. These morbidities frequently co-occur and may be associated with significant disability and impaired quality of life [8–11].

To address this issue, there is a growing need to develop interventions that can improve patient outcomes. An essential part of this process is developing a patient-centered “core outcome set” of measures that should be evaluated and reported in all clinical trials evaluating post-discharge outcomes in ICU survivors [12]. Core outcome sets allow more direct comparison of trial findings to draw more meaningful synthesis and conclusions on the effectiveness of interventions [13, 14]. However, the complex nature of outcomes after critical illness necessitates having an in-depth understanding of the problems ICU survivors face following hospital discharge. The patients’ perspective is essential in gaining this understanding. Qualitative research is a well-known methodology to collect and analyze in-depth information from patients, particularly on their health status that may help inform development of core outcome sets [15]. Recently, there has been an increase in qualitative research published on the patient perspective following critical illness [16–20]. In addition, qualitative research is increasingly being recognized as an important contribution to randomized controlled trials, with an important value in planning the trial, identifying important patient outcomes, and adding context to the trial findings [21, 22].

The Patient Reported Outcomes Measurement Information System (PROMIS) framework, developed by the US National Institutes of Health, represents a framework of health domains to report and understand patient-centered outcomes [23, 24]. Through this systematic review, we aim to identify and categorize important themes of patient-centered outcomes after critical illness from qualitative research, based on this framework of PROMIS domains. Findings from this study will help inform the development of core outcome sets for ICU survivors.

## Methods

Reporting of this systematic review was done according to the Preferred Reporting Items for Systematic Reviews (PRISMA) guidelines [25] where applicable. A written protocol and search strategy were developed, but were not publicly registered.

### Search strategy

Five electronic databases (PubMed, EMBASE, Cumulative Index of Nursing and Allied Health Literature (CINAHL), PsycINFO®, and the Cochrane Controlled Trials Registry (CENTRAL)) were searched for articles including any patient outcome assessments after hospital discharge in survivors of critical illness, based on a strategy developed for a scoping review (Additional file 1: Table S1) [14]. Within this broad search strategy, additional filters were applied to identify articles with qualitative findings, as defined in Additional file 1: Table S2 [26]. The search was conducted in June 2015, and was not limited by language or date of publication. A manual search of reference lists from all articles eligible for this systematic review was also performed.

### Study selection

Articles were eligible for inclusion in this systematic review if the study population was adults (>18 years old) and >50 % of them were discharged from the ICU, and if the study included qualitative findings focusing on patient outcomes after hospital discharge. Qualitative findings were defined as themes or quotes, or a combination of both, reported from patient/proxy interviews. To capture a broad range of qualitative research and maximize content for our thematic analysis, we included all qualitative research methodologies in eligible studies, including patient/proxy interviews, focus groups, and open-ended surveys [27]. To help ensure findings were generalizable to broad populations of ICU survivors, studies were excluded if they focused exclusively on a specific patient population (e.g., liver transplant) or specialty ICU (e.g., trauma or neurological ICUs), similar to prior published systematic reviews of ICU survivors [4, 6, 28, 29]. Using these eligibility criteria, trained reviewers (UN, SN, KN, and MDH) screened titles/abstracts and full-text articles in duplicate, using DistillerSR^©^ (2014; Evidence Partners, Ottawa, Canada; https://v2.systematic-review.ca). Disagreement regarding eligibility was resolved by consensus, after discussion with a third party (MNE, DMN, or VDD).

### Data extraction

Data were extracted sequentially by two trained research staff (AN and MDH) for each eligible article, with one reviewer extracting data and a second comparing the data against the original article. Extracted data included patient demographics, study type (e.g., focus groups, semi-structured interviews, ethnographic observations, etc.), and all qualitative themes identified and reported. In addition, a quality assessment of eligible studies was conducted, adapted from a previously published standardized framework [30] based on Cochrane guidelines [26] and the Critical Appraisal Skills Programme (http://www.casp-uk.net/#!casp-tools-checklists/c18f8). This quality assessment included appraisal of the rationale for qualitative methods, sample size, description of ineligible patients and those who chose not to participate in the study, use of a recording method, use of an interview guide, reporting coding reliability statistics, reporting number of coders per interview, reporting method of addressing of discrepancies, using a codebook, using a theory, and supporting themes by quotes.

### Data synthesis

All themes relevant to patient outcomes after hospital discharge were identified and extracted from all eligible studies. For studies that included patient and proxy outcomes, only themes that were specific to the patient were included. Through discussion and consensus among three reviewers (MNE, VDD, and MDH), these themes were coded and then categorized using the following main domains of the PROMIS framework [23, 24]: mental health conditions/symptoms (which encompasses cognitive outcomes), physical health, and social health including ability to participate in social roles and activities [31]—global satisfaction with life was added as a separate domain owing to its importance for survivors of critical illness.

## Results

### Study selection

Search results identified 2735 citations. After removing duplicates across databases, 2376 unique abstracts and titles were screened to meet the inclusion criteria, from which 459 full-text articles were reviewed, and 22 met eligibility criteria (Additional file 1: Figure S1).

### Study details

Table 1 presents details of the 22 eligible studies, conducted in 10 different countries. Only three (14 %) studies were conducted prior to the year 2000 [32–34]. A total of 17 (77 %) studies were longitudinal with a maximum of five follow-up time points, with 15 (68 %) of the studies involving patient report only and seven (32 %) studies involving both patient and caregiver/proxy reports. The majority of studies utilized open-ended patient/proxy interviews, while two (9 %) studies utilized focus groups. Data were collected exclusively in person in 16 (73 %) studies, with the remaining collected by telephone, mail, or Internet survey, or a combination of these methods.

**Table 1:** Study characteristics

Study	Country	Total number	Number of male patients (%)	Age (years)^a^	Number of time points	Type of interviewee	Outcomes focused on	Data collection method	Data collection mode
Russell [34]	Australia	298	–	–	1	Patient and caregiver/proxy	Patient	Telephone/in person	Interview
Maddox et al. [40]	Australia	5	2 (40 %)	60 [42–76]	NA	Patient and caregiver/proxy	Patient and caregiver/proxy	In person	Interview
Papathanassoglou and Patiraki [46]	Greece	8	3 (38 %)	–	3	Patient	Patient	In person	Interview
Williams [39]	UK	11	–	–	2	Patient	Patient	In person	Interview
Talisayon et al. [49]	Australia	5	4 (80 %)	50 (20)	NA	Patient	Patient	In person	Interview/mixed
Storli et al. [36]	Norway	10	4 (40 %)	46 [28–70]	NA	Patient	Patient	In person	Interview
Sawdon et al. [32]	UK	–	–	–	1	Patient and caregiver/proxy	Patient and caregiver/proxy	Telephone/in person	Interview
Ramsay et al. [43]	UK	20	11 (57 %)	61 (49–71)	1	Patient	Patient	In person	Interview
Prinjha et al. [51]	UK	34	20 (59 %)	52 (14)	NA	Patient	Patient	In person	Interview
Pattison et al. [50]	UK	22	8 (36 %)	59 (12.9)	2	Patient	Patient	Email interviews	Interview/mixed methods
Hall-Smith et al. [33]	UK	26	–	–	1	Patient	Patient	In person	Interview
Walker et al. [37]	UK	16	11(69 %)	43 (14.8)	1	Patient	Patient	In person	Focus groups
Ewens et al. [48]	Australia	18	9 (50 %)	[34–84]	3	Patient	Patient and caregiver/proxy	Mail	Interview
Deacon [47]	Multiple countries	35	5 (14 %)	48 (9.79)	NA	Patient	Patient	Online questionnaire	–
Czerwonka et al. [18]	Canada	5	3 (60 %)	–	5	Patient and caregiver/proxy	Patient	Telephone/in person	Interview
Corrigan et al. [35]	Sweden	14	3 (21 %)	52 [42–74]	2	Patient	Patient	In person	Interview
Chiang [41]	China	4	3 (75 %)	67.5 (9.86)	1	Patient and caregiver/proxy	Patient and caregiver/proxy	In person	Interview
Chahraoui et al. [45]	France	20	9 (45 %)	68 (8.5)	1	Patient	Patient	In person	Interview
Agård et al. [16]	Denmark	18	11(61 %)	55 (12.29)	2	Patient and caregiver/proxy	Patient	In person	Focus groups and interview
Adamson et al. [42]	Australia	6	4 (67 %)	64 [57–83]	1	Patient and caregiver/proxy	Patient	In person	Interview
Abdalrahim and Zeilani [38]	Jordan	18	7 (39 %)	53 (15.6)	1	Patient	Patient	In person	Interview
Ewens et al. [44]	Australia	1	1 (100 %)	37	2	Patient	Patient	In person	Interview

^a^Data presented as mean (standard deviation), as median (interquartile range), or as median [absolute range]

*NA* not applicable because the study was not longitudinal, – not reported

### Quality appraisal

The majority of eligible studies (86 %) described a rationale for using qualitative methods, developed a codebook (72 %), used theory (73 %), and had themes supported by quotations from the patient assessments (86 %). On the other hand, only a minority of studies described a rationale for the sample size (e.g., data saturation) (23 %), or discussed which patients were ineligible (36 %) or which chose not to participate (18 %). Seven studies (32 %) reported using ≥2 coders per interview (Additional file 1: Table S3).

### Thematic findings

**Fig. 1:**
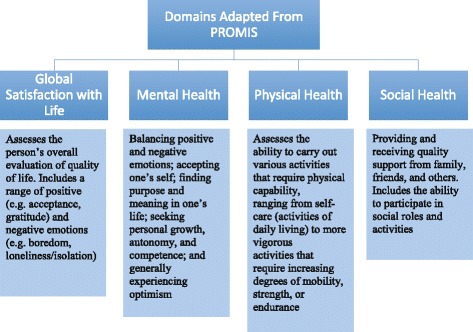
Domains adapted from the Patient Reported Outcomes Measurement Information System (PROMIS). Definitions adapted from http://www.nihpromis.com/measures/domainframework1; and https://www.healthypeople.gov/2020/topics-objectives/topic/health-related-quality-of-life-well-being

Thematic findings were represented in five major domains adapted from PROMIS; global satisfaction with life, mental health, physical health, social health, and ability to participate in social roles and activities (Fig. 1). Interestingly, older studies more often focused on mental health (mainly depression and post-traumatic stress disorder) [33–35], while physical health, global satisfaction with life, and social health appeared more often in newer studies [18, 36–38].

#### Global satisfaction with life

The domain of global satisfaction with life reflected themes that represented global patient functioning and were not specific to a particular PROMIS domain. These themes include a range of positive and negative emotions (Table 2). In 10 studies, patients described positive emotions related to their overall view of life in response to critical illness, such as finding a new source of motivation and strength [32, 36–44]. Acceptance was another common theme, emerging from seven studies, whereby patients described how their feelings transformed from anger and denial to accepting the consequences of their illness and trying to move on [34, 36, 38, 40–42, 45]. In addition, gratitude emerged from five studies, where patients felt thankful for surviving critical illness, with deeper appreciation of the value of life [36–39, 46]. Independence was identified in four studies, with patients commenting on the value of being able to do basic tasks without assistance [16, 18, 40, 41]. On the contrary, patients in four studies commented on having negative emotions related to satisfaction with life; themes generated from these studies included boredom [37], loneliness/isolation [38, 44], and feeling that they wish they had not lived [35].

**Table 2:** Qualitative themes of satisfaction with life

Theme	Example quote(s)
Positive outlook	1. “I have got such a different outlook on life … I’ll have the odd day where I will dwell a bit … but everyday is a new day and its a beautiful day … I’m a different person.” 2. “One might well say that it has been for me, even though it sounds strange—a good experience. And the fact that I continually find out more about the emotionally strong memories I have, actually provides me with insight into myself!”
Acceptance	“You live with what you’ve got—–that is the attitude I took. Alright I’m sick and I’ve got this and I’ve got that. I can’t do this and maybe I won’t be able to do that, so you adjust and you move on.”
Gratitude	1. “I try to cram in as much living as I can, because it could all end tomorrow …” 2. “I look out from the window and I see people walking and I cry out of joy, because I am alive … and I had never realized that before …”
Independence	“It's great in a way [being back at home) because you have to do things on your own. And then you start getting stronger and you take your rest breaks, and you do it again, and you do get assistance, you would need the assistance like for grocery shopping and stuff like that. But as time goes by you start getting stronger.”
Boredom	“… that’s the worst thing about coming out of hospital, sitting doing nothing …”
Loneliness/isolation	“I do not enjoy being with people, usually I keep silent. I don’t want to be with them or to share their talks … They asked a lot … They asked about being in the unit … I feel that they sympathize with me … I do not want to remember that time.”
Wish they had not lived	“Sometimes in the evenings I’ve thought to myself … ‘hell, it would have been better to have slipped away’ … then you feel nothing.”

#### Mental health

Mental health was a commonly identified domain (Table 3). The theme of post-traumatic stress disorder symptoms was identified in eight studies, emerging from the experience of having recurrent dreams/nightmares or flashbacks related to the ICU stay [33, 34, 38, 45, 47, 48], as well as the negative emotions that arose when reminded of their critical illness (e.g., driving near a hospital, or even seeing/hearing sirens or alarms on radio or television [35, 36]). Similarly, anxiety symptoms was a common theme in eight studies, where patients described constant fear [37, 49, 50], worrying [35, 42, 45], and panic attacks [36, 45]. Depression symptoms emerged from six studies, where patients commented on “not seeing the point of anything” [51], inability to look forward [33], feeling disappointed [50], depressed [45], and crying uncontrollably for no reason [35, 44, 51]. Irritability/anger was less commonly described, in just three studies, whereby patients reported frustration with disabilities [50] and deformities [37], as well as feeling “on guard” [45]. Overall emotional functioning was described in seven studies with themes that did not clearly map onto a well-described psychological disorder, including general mood changes [33, 47, 49, 50], inability to cope with stress [33, 35, 51], and loss of motivation [37].

**Table 3:** Qualitative themes of mental health conditions/symptoms

Theme	Example quote(s)
Post-traumatic stress disorder symptoms	1. “I often wake up terrified because I had dreams of being in the unit with all the sounds and noises of machines. Even when I’m awake and with people, many things remind me of the unit, people talking, and images from the TV.” 2. “It’s enough that someone says something on the radio, mentions the term intensive care or something, or when you watch teletext or TV and that word crops up, or there’s a nurse working with … at the ICU, ugh, then I feel exactly like I did then.”
Anxiety	1. “I was having these major panic attacks when I arrived in Cardiology and I’m still having them, even now that I’m home, it’s often in the evening, every evening I get really panicky.” 2. “Previously I was a harmonious, calm, positive and stable person, now things are different … I’m more insecure and nervous, which I wasn’t before.”
Depression	“There are still days even, what are we six, seven months on now, yeah I just couldn't see the point of anything … in my mind I was thinking ‘Well what's the point of it, we're all going to die anyway?’ And I needed to speak to, I went back and spoke to the nurse consultant on ICU and she explained it’s perfectly normal. And that helped, once she said to me, ‘Loads of people feel like that when they come out of intensive care and you need to be kind and give yourself a bit of time, it will pass.’”
Irritability/anger	1. “I am now beginning to get frustrated with the things I still cannot do … I am finding it increasingly difficult mentally to cope with my recovery.” 2. “After a while, well, I’m not a nasty person, but I’m more on my guard now …; I’m more irritable, more nervous.”
General emotional functioning	1. “I cried for the slightest reason … it … only took the smallest setback.” 2. “You could never imagine the emotional side even if you were prepared … emotionally it was a rollercoaster.”

#### Physical health

Physical health was another common domain (Table 4). Mobility was an important theme highlighted in eight studies; patients’ impaired mobility following discharge had a major impact on their overall wellbeing [16, 18, 40, 42, 47], due to the impact on their ability to complete daily activities or return to work. This was highlighted by patients reporting about the importance of regaining mobility, even if only partially [16, 18, 37, 38, 40]. Also important was regaining the capacity to perform activities of daily living, identified in five studies, which appears to be important for both patients [16, 48, 50] and caregivers [18, 41]. Fatigue and subjective feeling of weakness were identified in four studies [16, 18, 33, 50]. Sleep disturbances emerged as a recurring theme in three studies, where patients complained of nightmares [45] and recurrent sleep interruptions [33, 35, 45]. Other less common themes were disturbances in appetite [45, 50], sensory changes [18, 37], and muscle weakness [16].

**Table 4:** Qualitative themes of physical health

Theme	Example quote(s)
Mobility	1. “I can move now, before, I thought I will stay handicapped all my life. But never mind, I feel grateful just to have the ability to walk again …” 2. “Then I had to try to get up with a walker and I just couldn’t. I couldn’t even hold my head. I wasn’t able to do anything.”
Activities of daily living	1. “My day-to-day life is anything but normal. I want to be able to cook, clean and do the gardening, walk to the shops … recovery has been reasonable.” 2. “For the first couple of weeks, we were sort of … doing things like getting his clothes and organizing his shower and all that sort of thing. I practically was just running around doing little bits for him, organizing what tablets he had to take and all this sort of thing.”
Fatigue	1. “I probably went too far. I mean, I was at home and tried to arrange that my husband didn’t need to come home and do things. But then I was tired and couldn’t handle it anyway.” 2. “I need an afternoon nap, sometimes two …”
Appetite	“Now it’s going ok again, I’m eating well, and I’m sleeping so well! I feel better, even better than before …, a little frustration at not having an appetite and my insides not really knowing where they should be ….”
Sensory changes	“I also have double vision … I can’t read … I can’t even watch telly … it’s like being in prison.”
Muscle weakness	“The most difficult bit was … I felt it took forever before I regained my strength. I just deposited my physical strength at the hospital and I still feel it. I mean, I don’t feel I am up to my usual strength yet … I feel that I need more strength to open the lid of a jar of jam. I was actually quite strong before I got sick.”
Sleep disturbances	1. “I slept so badly, I had these awful dreams, really horrible …”; “It’s incredible to wake up all of a sudden only to find that, well, everything’s OK, and you wake up anyway just to check …” 2. “I’m sleeping really badly, I wake up often, I only sleep in small bits, it’s true that my sleep is not doing me good.”

#### Social health

Social health was the fourth identified major domain (Table 5). Changes in friends or family relationships was a recurring theme; these changes were described in the form of patients not enjoying being with people anymore [38], feeling like a burden [35], feeling annoyed as a result of new restrictions enacted by family, or feeling “not needed” by family [38]. On the contrary, two studies highlighted how patients saw improvement in social functioning after critical illness, such as recognizing the importance of family in a crisis situation [36] and getting to see friends as an important part of the recovery process [45].

**Table 5:** Qualitative themes of social health and ability to participate in social roles and activities

Theme	Example quote(s)
Social roles, activities, or relationships	
Changes in friends or family relationships	1. “I’m happy being back home, but I feel that my kids do not need me anymore. They use to take my opinion in every aspect of their lives, I can’t find this anymore. Now they consult their mother, and act as if I’m still in hospital.” 2. “… since the accident I don’t socialize as much as I used to … If you go out with friends you know, two years down the line, they don’t want to be talking about your illness.”
Ability to participate in social roles and activities	
Hobbies	“It was important for me to get back to a normal life, to paint or put up wallpaper or other stuff like that …”
Disability	“I was a mechanic … I can’t do it now. I’m not allowed to drive a car or get on a plane, they won’t let me do anything.”

#### Ability to participate in social roles and activities

The domain of ability to participate in social roles and activities, which is categorized under social health according to the PROMIS framework, was the fifth major domain (Table 5). Getting back to previous hobbies was an important source of motivation during recovery [42, 45]. On the contrary, the inability to perform prior activities was regarded as a form of disability and distress [37, 42]. This theme was often described in connection with physical and emotional functioning and deeply impacted survivors’ overall satisfaction with life. The change in work status was often a major issue for patients as they either celebrated their ability to return to work or had to reconcile a major life change if they were unable to return to work.

## Discussion

In this systematic review of qualitative studies of general critical illness survivors, themes related to outcomes after hospital discharge were extracted from studies and then categorized using the PROMIS domains of patient outcomes: global satisfaction with life, mental health, physical health, social health, and ability to participate in social roles and activities. Our findings indicate that ICU survivors experience impairments unique to their critical illness across all domains of PROMIS.

PICS, developed as part of a 2010 stakeholder conference based on existing literature and discussion/consultation among multidisciplinary experts and patient/family representatives [7], is an effort to raise awareness of the range of sequelae faced by survivors of critical illness. PICS specifically considered physical, cognitive, and mental health impairments [7]. The findings of this systematic review of qualitative studies clearly support the mental health and physical impairments that are part of PICS. However, none of the eligible studies in our systematic review explicitly reported a theme related to cognitive functioning, in contrast to the wealth of data reported in quantitative studies [52, 53]. This may be a limitation of previous qualitative research, or a result of the study selection criteria for this systematic review.

In addition to affirming the domains included in PICS, our findings highlight the importance of a social health domain. The theme of social health includes social functioning and ability to participate in social roles and activities. According to the PROMIS definition, “Social health encompasses participation in activities with others, carrying out one’s usual roles and responsibilities, and relationships and connections with important others” [54, 55]. This theme was less frequently reported than themes related to global satisfaction with life and to mental and physical health. Some quantitative studies have demonstrated significantly lower quality of life in ICU survivors compared with the general population [10, 56–58], which may persist over time [58, 59]. Social health may be an important contributing factor to impaired quality of life [60]. However, characterizing the social impact of critical illness can be challenging using quantitative tools [61], and this may be a reason for not including social health in quantitative studies evaluating impairments following critical illness [3]. Social function is only assessed by two questions in the Short Form-36 version 2 health survey (SF-36 v2) and is not assessed by the EQ-5D survey, two of the most common tools used to quantify quality of life [14, 62]. A study of 980 ICU survivors showed significant correlation between social integration level (measured by the Availability of Social Integration instrument) and SF-36 quality-of-life outcomes, with this correlation not present in matched controls [63]. Although the PROMIS outcome measures for social health have not yet been used in ICU survivors, findings from our systematic review highlight this domain for consideration in future research (Table 5).

Domains related to mental health and physical health were well represented in our findings. These domains are also well recognized by quantitative studies of ICU survivors, which demonstrate high prevalence of symptoms of depression [64] and post-traumatic stress disorder [4], as well as physical disability [5, 8, 9]. Our findings echo those from quantitative studies, highlighting the high prevalence of these sequelae and their great impact on patients’ overall wellbeing.

Our findings also highlight the range of positive emotions patients may experience following critical illness, which included positive outlook, acceptance, gratitude, and independence. These findings demonstrate the importance of appropriate coping mechanisms for patients following critical illness to promote a positive life outlook. The importance of coping in response to life events and related stress is well recognized [65], and positive coping skills have a strong association with improved quality of life in other patient populations [66, 67]. Hence, strategies to improve coping skills may help improve patient outcomes following critical illness [68, 69].

Our critical appraisal of eligible studies showed that most of them used rigorous methods for recording and coding qualitative data. However, there were important gaps related to a lack of reporting on patient selection, sample size justification, and use of a structured interview guide (Additional file 1: Table S2). Utilizing a standardized framework for reporting qualitative research is essential for comparing studies to draw meaningful conclusions [26]. Furthermore, it is critical to assist with replication and transparency of methods.

These findings can help inform future research aimed at ensuring a patient-centered approach in evaluating the impact of critical illness, as emphasized in recently developed initiatives such as PROMIS and the Patient-Centered Outcomes Research Institute [70]. The findings of this systematic review also can contribute to ensuring appropriate patient-centered domains are included in a core outcome set for evaluating ICU survivors’ outcomes after hospital discharge [12, 71–73] and may be useful to consider in future revisions of PICS.

There are potential limitations to this systematic review. First, it is important to note that many of the studies included in our review did not report methods related to appropriate patient sampling, data saturation, or inter-rater comparisons; therefore, our inferences may be limited by these issues. Second, the eligible studies were heterogeneous in the methods used to collect and analyze the qualitative data, which might have limited our ability to synthesize findings across studies. However, our data represent 10 different countries, providing some evidence regarding the shared global impact of critical illness. Given that our study used the PROMIS framework to categorize themes, our ability to perform a meta-synthesis of the findings may have been limited [74]. However, we used the PROMIS framework to categorize themes since it is frequently used to understand patient outcomes across many chronic diseases [54]. Lastly, all attempts were made to identify and include all relevant studies; however, potentially relevant studies may have been inadvertently omitted.

## Conclusion

This systematic review of qualitative studies evaluating survivors of critical illness after hospital discharge demonstrates that some may experience positive emotions (e.g., acceptance, gratitude, and positive outlook). However, many survivors face a wide range of mental, physical, and social sequelae with impaired life satisfaction. These findings are important in helping focus on patient-centered outcomes for studies aimed at improving the status of survivors of critical illness, and in refining randomized controlled trials in the field by identifying patients’ perspectives on outcomes and considering potential interventions to address these needs.

## Additional file

Additional file 1: Is Table S1 presenting the search strategy, Table S2 presenting the published versions of methodological filters for retrieving qualitative research, Table S3 presenting the quality appraisal of reporting of qualitative studies, and Figure S1 showing a flow diagram for identifying eligible studies. (DOC 176 kb)

## Abbreviations

ICU: Intensive care unit
PICS: Post-intensive care syndrome
PRISMA: Preferred Reporting Items for Systematic Reviews
PROMIS: Patient Reported Outcomes Measurement Information System
SF-36 v2: Short Form-26 version 2 health survey
