# Supplementary material for: Patient outcomes after critical illness: a systematic review of qualitative studies following hospital discharge
Source: Crit Care. 2016 Oct 26;20:345. doi: 10.1186/s13054-016-1516-x (PMC5080744; doi:10.1186/s13054-016-1516-x)
Supplement: Additional file 1: — Is Table S1 presenting the search strategy, Table S2 presenting the published versions of methodological filters for retrieving qualitative research, Table S3 presenting the quality appraisal of reporting of qualitative studies, and Figure S1 showing a flow diagram for identifying eligible studies. (DOC 176 kb) [file 13054_2016_1516_MOESM1_ESM.doc]

**Patient Outcomes after Critical Illness: A Qualitative Systematic Review**

**Additional file 1**

Mohamed D. Hashem, MD ^1,2^

Aparna Nallagangula, MBBS ^1,2^

Swaroopa Nalamalapu, MBBS ^1,2^

Krishidhar Nunna, MBBS ^1,2^

Utkarsh Nausran^1^

Karen A. Robinson, MSc, PhD ^3^

Victor D. Dinglas, MPH ^1,2^

Dale M. Needham, FCPA, MD, PhD ^1,2,4^

Michelle N. Eakin, PhD ^1,2^

**Table E3. Quality Table E3. Quality appraisal of reporting of qualitative studies**

**appraisal of reporting of qualitative studies**

**Table S**1. Search Strategy

| **PubMed** | (intensive care[tiab] OR "intensive care"[MeSH Terms] OR intensive therapy[tiab] OR high dependency[tiab] OR critical care[tiab] OR "critical care"[MeSH Terms] OR intermediate care[tiab] OR step-up care[tiab] OR step-down care[tiab] OR respiratory distress syndrome[tiab] OR acute lung injury[tiab]) AND (outcome measure[tiab] OR "outcome assessment (health care)"[MeSH Terms] OR follow-up[tiab] OR "follow-up studies"[MeSH Terms] OR health status[tiab] OR "health status"[MeSH Terms] OR functional status[tiab] OR clinical outcome[tiab]) AND (organ failure[tiab] OR "multiple organ failure"[MeSH Terms] OR organ dysfunction[tiab] OR sequelae[tiab] OR quality of life[tiab] OR "quality of life"[MeSH Terms] OR impairment[tiab] OR morbidity[tiab] OR "morbidity"[MeSH Terms]) NOT (animals[mh] NOT humans[mh])or “Patient outcome assessment” [MeSH Terms} |
| --- | --- |
| **Embase** | ('intensive care'/exp OR 'intensive care':ti,ab OR 'intensive therapy':ti,ab OR 'high dependency':ti,ab OR 'critical care':ti,ab OR 'critical care':ti,ab OR 'intermediate care':ti,ab OR 'step-up care':ti,ab OR 'step-down care':ti,ab OR 'respiratory distress syndrome':ti,ab OR 'acute lung injury':ti,ab) AND ('outcome measure':ti,ab OR 'outcome assessment'/exp OR 'follow up':ti,ab OR 'follow up'/exp OR 'health status':ti,ab OR 'health status'/de OR 'functional status':ti,ab OR 'clinical outcome':ti,ab) AND ('organ failure':ti,ab OR 'multiple organ failure'/exp OR 'organ dysfunction':ti,ab OR 'multiple organ failure':ti,ab OR 'sequelae':ti,ab OR 'quality of life'/exp OR 'quality of life':ti,ab OR 'impairment':ti,ab OR 'morbidity':ti,ab OR 'morbidity'/exp) NOT ('animal'/exp NOT ('animal'/exp AND 'human'/exp)) |
| **CINAHL** | (MH "intensive care units+" OR "intensive care" OR "intensive therapy" OR "high dependency" OR MH "critical care" OR "critical care" OR "intermediate care" OR "step-up care" OR "step-down care" OR "respiratory distress syndrome" OR "acute lung injury") AND ("outcome measure" OR MH "outcome assessment" OR "follow up" OR MH "prospective studies" OR MH "health status" OR "health status" OR "functional status" OR "clinical outcome") AND ("organ failure" OR "multiple organ failure" OR "organ dysfunction" OR MH "multiple organ dysfunction syndrome" OR "sequelae" OR "quality of life" OR MH "quality of life" OR "impairment" OR "morbidity" OR MH "morbidity") |
| **PsycINFO** | (DE "intensive care" OR "intensive care" OR "intensive therapy" OR "high dependency" OR "critical care" OR "intermediate care" OR "step-up care" OR "step-down care" OR "respiratory distress syndrome" OR "acute lung injury") AND ("outcome measure" OR "outcome assessment" OR "follow up" OR DE "Followup Studies" OR "health status" OR "functional status" OR "clinical outcome") AND ("organ failure" OR "multiple organ failure" OR "organ dysfunction" OR "sequelae" OR "quality of life" OR DE "quality of life" OR "impairment" OR DE "morbidity" OR "morbidity") |
| **Mesh Terms** | Intensive Care MeSH Terms:  intensive care  critical care  Intensive Care Keywords:  "intensive care":ti,ab,kw OR "intensive therapy":ti,ab,kw OR "high dependency":ti,ab,kw OR "critical care":ti,ab,kw OR "intermediate care":ti,ab,kw OR "step-up care":ti,ab,kw OR "step-down care":ti,ab,kw OR "respiratory distress syndrome":ti,ab,kw OR "acute lung injury":ti,ab,kw  Outcomes MeSH Terms:  outcome assessment (health care)  follow-up studies  health status  Patient Outcome Assessment  Outcomes Keywords:  "outcome measure":ti,ab,kw OR "follow-up":ti,ab,kw OR "health status":ti,ab,kw OR "functional status":ti,ab,kw OR "clinical outcome":ti,ab,kw  Specific Outcomes MeSH Terms:  multiple organ failure  morbidity  quality of life  Specific Outcomes Keywords:  " organ failure":ti,ab,kw OR "organ dysfunction":ti,ab,kw OR "sequelae":ti,ab,kw OR "quality of life":ti,ab,kw OR "impairment":ti,ab,kw OR "morbidity":ti,ab,kw |

**Table S2**. Published versions of methodological filters for retrieving qualitative research

| **Database** | **Filter** | **Suggested Filter from article** |
| --- | --- | --- |
| CINAHL | Wilczynski NL, Marks S, Haynes RB. Search strategies for identifying qualitative studies in CINAHL. *Qualitative Health Research* 2007;**17**(5):705-10. | exp interviews (explode) |
| EMBASE | Walters LA, Wilczynski NL, Haynes RB; Hedges Team. Developing optimal search strategies for retrieving clinically relevant qualitative studies in EMBASE. *Qualitative Health Research* 2006, **16**(1):162-8. | Qualitative.tiab  OR qualitative study.tiab. |
| MEDLINE | Important note: MEDLINE strategies reported below precede introduction of the MeSH Heading ‘Qualitative Research’ (2003). This term should be added to these strategies.  Wong SS, Wilczynski NL, Haynes RB. Developing optimal search strategies for detecting clinically relevant qualitative studies in MEDLINE. *Medinfo* 2004;**11**(1):311-6. | qualitative.tiab. OR  themes.tiab.  MeSH heading ‘Qualitative Research” |
| PsycINFO | McKibbon KA, Wilczynski NL, Haynes RB. Developing optimal search strategies for retrieving qualitative studies in PsycINFO. *Evaluation & the Health Professions* 2006; **29**(4):440-54. | qualitative:.tiab  OR themes.tiab. |

| **Study** | **Rationale for qualitative methods stated?** | **Rationale for sample size stated?** | **Described who was ineligible?** | **Described who chose not to participate?** | **Was recording used for data collection?** | **Was there an interview guide?** | **Any reliability/validity stats reported?** | **No. of coders per interview** | **Reported addressing of discrepancies?** | **Was codebook developed?** | **Was theory used?** | **Were themes supported by quotes?** |
| --- | --- | --- | --- | --- | --- | --- | --- | --- | --- | --- | --- | --- |
| Russell [1] | + | - | - | - | - | - | - | - | - | + | - | + |
| Maddox, *et al* [2] | + | - | - | - | + | - | - | - | + | ? | ? | + |
| Papathanassoglou, *et al* [3] | ? | - | - | - | - | - | - | - | + | + | + | + |
| Williams [4] | + | - | - | - | - | - | - | - | - | + | + | - |
| Talisayon, *et al* [5] | + | - | + | - | + | - | - | 2 | + | - | + | - |
| Storli, *et al* [6] | + | - | + | - | + | ? | - | - | + | + | + | + |
| Sawdon, *et al* [7] | + | - | - | - | - | - | - | - | - | - | + | + |
| Ramsay, *et al* [8] | + | - | + | - | + | + | - | - | - | + | + | + |
| Suman Prinjha, *et al* [9] | + | + | - | - | + | - | - | 2 | - | + | + | + |
| Pattison, *et al* [10] | + | - | + | - | + | ? | - | - | - | + | + | + |
| Hall-Smith, *et al* [11] | + | - | + | + | + | - | - | 2 | - | - | - | + |
| Walker, *et al* [12] | + | - | - | - | + | + | - | - | - | + | + | + |
| Ewens *et al* [13] | + | - | - | - | - | + | - | - | - | + | - | + |
| Deacon [14] | + | - | - | - | + | - | - | 1 | - | - | + | + |
| Czerwonka, *et al* [15] | + | + | + | - | + | + | - | 3 | + | + | + | + |
| Corrigan, *et al* [16] | + | - | - | - | + | - | - | - | - | + | + | + |
| Chiang [17] | + | + | - | + | + | - | - | - | + | + | + | + |
| Chahraoui, *et al* [18] | + | + | + | - | + | - | - | 3 | + | + | - | + |
| Agard, *et al* [19] | - | - | + | - | + | + | - | 2 | - | + | + | + |
| Adamson, *et al* [20] | - | + | - | + | + | + | - | - | + | + | - | + |
| Abdalrahim, *et al* [21] | + | - | - | + | + | ? | - | 2 | + | + | + | + |
| Ewens, *et al* [22] | + | - | - | - | + | - | - | 1 | - | - | + | - |

**Table S3. Quality appraisal of reporting of qualitative studies**

**Figure S1. Flow diagram for identifying eligible studies.**

Abbreviations: CINAHL, Cumulative Index of Nursing and Allied Health Literature; CENTRAL, Cochrane Controlled Trials Registry

* Total number exceeds the number of citations excluded because citations could be excluded for more than one reason

**Retrieved**

2735

**Electronic Databases**

Pubmed**765**Embase**864**CINAHL**836**PsycInfo**219**Cochrane**51**

**Abstract Review**

2376

**Duplicates**

748

**Full Article Review**

459

**Excluded**

1917

Did not meet inclusion criteria for full text review

**Eligible Articles**

22

**Excluded**

437

Reasons for Exclusion*

184 – Does not include qualitative results

84 – Only describes outcomes in-hospital

80 – Study population does not include ICU patients

52 – Focus on specialized patient/ICU

47 – Abstract only

44 – No original research data

13 – Focus on proxy/caregiver

12 – Other reason

5 – Duplicate

2 – Less than 5 patients in sample

**Hand Search**

389

grderte

Reference List

1. Russell S. An exploratory study of patients’ perceptions, memories and experiences of an intensive care unit. J. Adv. Nurs. 1999;29:783–91.

2. Maddox M, Dunn SV, Pretty LE. Psychosocial recovery following ICU: experiences and influences upon discharge to the community. Intensive Crit. Care Nurs. Off. J. Br. Assoc. Crit. Care Nurses. 2001;17:6–15.

3. Papathanassoglou EDE, Patiraki EI. Transformations of self: a phenomenological investigation into the lived experience of survivors of critical illness. Nurs. Crit. Care. 2003;8:13–21.

4. Williams SL. Recovering from the psychological impact of intensive care: how constructing a story helps. Nurs. Crit. Care. 2009;14:281–8.

5. Talisayon R, Buckley T, McKinley S. Acute post-traumatic stress in survivors of critical illness who were mechanically ventilated: a mixed methods study. Intensive Crit. Care Nurs. Off. J. Br. Assoc. Crit. Care Nurses. 2011;27:338–46.

6. Storli SL, Lindseth A, Asplund K. A journey in quest of meaning: a hermeneutic-phenomenological study on living with memories from intensive care. Nurs. Crit. Care. 2008;13:86–96.

7. Sawdon V, Woods I, Proctor M. Post-intensive care interviews: implications for future practice. Intensive Crit. Care Nurs. Off. J. Br. Assoc. Crit. Care Nurses. 1995;11:329–32.

8. Ramsay P, Huby G, Thompson A, Walsh T. Intensive care survivors’ experiences of ward-based care: Meleis’ theory of nursing transitions and role development among critical care outreach services. J. Clin. Nurs. 2014;23:605–15.

9. Prinjha S, Field K, Rowan K. What patients think about ICU follow-up services: a qualitative study. Crit. Care Lond. Engl. 2009;13:R46.

10. Pattison N, O’Gara G, Rattray J. After critical care: patient support after critical care. A mixed method longitudinal study using email interviews and questionnaires. Intensive Crit. Care Nurs. Off. J. Br. Assoc. Crit. Care Nurses. 2015;31:213–22.

11. Hall-Smith J, Ball C, Coakley J. Follow-up services and the development of a clinical nurse specialist in intensive care. Intensive Crit. Care Nurs. Off. J. Br. Assoc. Crit. Care Nurses. 1997;13:243–8.

12. Walker W, Wright J, Danjoux G, Howell SJ, Martin D, Bonner S. Project Post Intensive Care eXercise (PIX): A qualitative exploration of intensive care unit survivors’ perceptions of quality of life post-discharge and experience of exercise rehabilitation. J. Intensive Care Soc. 2015;16:37–44.

13. Ewens B, Chapman R, Tulloch A, Hendricks JM. ICU survivors’ utilisation of diaries post discharge: a qualitative descriptive study. Aust. Crit. Care Off. J. Confed. Aust. Crit. Care Nurses. 2014;27:28–35.

14. Deacon KS. Re-building life after ICU: a qualitative study of the patients’ perspective. Intensive Crit. Care Nurs. Off. J. Br. Assoc. Crit. Care Nurses. 2012;28:114–22.

15. Czerwonka AI, Herridge MS, Chan L, Chu LM, Matte A, Cameron JI. Changing support needs of survivors of complex critical illness and their family caregivers across the care continuum: a qualitative pilot study of Towards RECOVER. J. Crit. Care. 2015;30:242–9.

16. Corrigan I, Samuelson KAM, Fridlund B, Thomé B. The meaning of posttraumatic stress-reactions following critical illness or injury and intensive care treatment. Intensive Crit. Care Nurs. Off. J. Br. Assoc. Crit. Care Nurses. 2007;23:206–15.

17. Chiang VCL. Surviving a critical illness through mutually being there with each other: a grounded theory study. Intensive Crit. Care Nurs. Off. J. Br. Assoc. Crit. Care Nurses. 2011;27:317–30.

18. Chahraoui K, Laurent A, Bioy A, Quenot J-P. Psychological experience of patients 3 months after a stay in the intensive care unit: A descriptive and qualitative study. J. Crit. Care. 2015;30:599–605.

19. Agård AS, Egerod I, Tønnesen E, Lomborg K. Struggling for independence: a grounded theory study on convalescence of ICU survivors 12 months post ICU discharge. Intensive Crit. Care Nurs. Off. J. Br. Assoc. Crit. Care Nurses. 2012;28:105–13.

20. Adamson H, Murgo M, Boyle M, Kerr S, Crawford M, Elliott D. Memories of intensive care and experiences of survivors of a critical illness: an interview study. Intensive Crit. Care Nurs. Off. J. Br. Assoc. Crit. Care Nurses. 2004;20:257–63.

21. Abdalrahim MS, Zeilani RS. Jordanian survivors’ experiences of recovery from critical illness: a qualitative study. Int. Nurs. Rev. 2014;61:570–7.

22. Ewens BA, Hendricks JM, Sundin D. Never ending stories: visual diarizing to recreate autobiographical memory of intensive care unit survivors. Nurs. Crit. Care. 2014;
